# The MRI-guided two adaptive brachytherapy fractions versus one adaptive brachytherapy fraction in one application for the cervical cancer: a retrospective study

**DOI:** 10.1186/s13014-023-02237-0

**Published:** 2023-03-06

**Authors:** Shanshan Song, Dongmei Han, Ning Zhang, Zhuang Mao, Xin Guo, Guanghui Cheng

**Affiliations:** Department of Radiation Oncology, China-Japan Union Hospital of Jilin University, No.126 Xiantai Street, Changchun, China

**Keywords:** Cervical cancer, IGABT, MRI, Intarcavitary/interstitial brachytherapy, Clinical outcome, Treatment-related complication

## Abstract

**Purpose:**

This study retrospectively compared the clinical and toxicity outcomes for the cervical cancer of the MRI-guided two adaptive brachytherapy (IGABT) fractions versus one IGABT fraction in one application.

**Methods:**

One hundred and twenty patients with cervical cancer received external beam radiotherapy combined with or without concurrent chemotherapy, which was followed by the IGABT. The IGABT in 63 patients had one IGABT in each application (Arm 1), while in the other 57 patients, at least one treatment was two continuous IGABT every other day in one application (Arm 2). Clinical outcomes including overall survival (OS), cancer specific survival (CSS), progression free survival (PFS), local control (LC) were analyzed. Brachytherapy-related toxicities were evaluated, which included pain, dizziness, nausea/vomiting, fever/infection, blood loss during the removal of applicator and needles, the deep venous thrombosis, and other acute toxicities. The Common Terminology Criteria for Adverse Events (CTC-AE 5.0) was used to evaluate the incidence and severity of toxicities of the urinary system, lower digestive system, and reproduction system. Kaplan–Meier and the Log-rank test were used to analyze the clinical outcomes.

**Results:**

The median follow-up time of the patients in Arm 1 and Arm 2 was 23.5 and 12.0 months, respectively. The overall treatment time was significantly shorter in Arm 2 than Arm 1 (60 vs. 64 d; *P* = 0.017). The OS, CSS, PFS, and LC in Arm1 and Arm 2 was 77.8% vs. 86.0% (*P* = 0.632), 77.8% vs. 87.7% (*P* = 0.821), 68.3% vs. 70.2% (*P* = 0.207), and 92.1% vs. 94.7% (*P* = 0.583), respectively. The highest NRS of the pain during brachytherapy waiting period (2.22 ± 1.84 vs. 3.02 ± 1.65; *P* < 0.001) and at the time of the removal of the applicator (4.69 ± 1.49 vs. 5.30 ± 1.18; *P* < 0.001) in the patients who received one hybrid intracavitary and interstitial brachytherapy (IC/ISBT) in one application and two continuous IC/ISBT every other day in one application were significantly different. So far four patients with grade 3 late toxicities have been reported.

**Conclusions:**

The findings of this study demonstrated that the two continuous IGABT every other day in one application is a logistically applicable, safe, and effective treatment strategy that could shorten the overall treatment time and reduce the medical cost, comparing with the one IGABT in one application.

**Supplementary Information:**

The online version contains supplementary material available at 10.1186/s13014-023-02237-0.

## Introduction

External beam radiotherapy (EBRT) in combination with brachytherapy (BT) is the common treatment approach for cervical cancer, of which the BT is a critical component of cervical cancer treatment [1]. In recent years, image-guided adaptive brachytherapy (IGABT) has been widely applied for cervical cancer. The commonly used image-guided techniques for the IGABT include CT, MRI, and ultrasound. Due to the high resolution of MRI on the soft tissues, the MRI has a substantial advantage in contouring the target area for the BT and makes the calculation of doses for the image-defined pelvic organs at risk (OARs) more accurate. As a consequence, the treatment efficacy is improved substantially, while the irradiations on the OARs, as well as its toxic effects, are reduced.

Previous studies have demonstrated that the overall treatment time (OTT) of > 7–8 weeks significantly influenced the treatment efficacy that was manifested by the reduction of the local control rate (LC) of tumor and overall survival (OS) [2]. Currently, when the EBRT is performed in combination with the BT, the BT is generally carried out 4–6 times, with 5–7 Gy in each time [3]. The BT is not recommended when the dose of EBRT is less than 40–45 Gy. In some patients, the OTT is prolonged to more than 7–8 weeks due to various causes. To shorten the OTT, two BT fractions could be performed every week for the patients receiving intracavitary brachytherapy (ICBT) after the EBRT; while for the patients receiving hybrid intracavitary and interstitial (IC/IS) BT, two BT fractions could be performed in one application to reduce the risk from the repeated general anesthesia and multiple invasive procedures within a short term [2].

This study aimed to investigate the treatment efficacy, BT-related toxicity, irradiation-related acute toxicity, and late toxicity of the MRI guided one high dose rate (HDR) BT fraction in one application and two continuous HDR-BT fractions every other day in one application, and to compare the two treatment models.

## Materials and methods

### Patients features

The data of 120 patients with the IB_2_-IVB (FIGO 2009) cervical cancer that were treated in our institute between November 2017 and October 2020 were retrospectively analyzed. All the patients had histopathologically proven adenocarcinoma, squamous carcinoma, or adenosquamous carcinoma, and were treated with the pelvic EBRT ± concurrent platinum-based chemotherapy and four fractions of ICBT or IC/ISBT with each dose of 7 Gy. The patients with a history of pelvic radiotherapy were excluded. The 63 patients that were hospitalized before October 2019 received one BT fraction in each application for the treatment (Arm 1). The 57 patients that were hospitalized after October 2019 received at least one time of two continuous BT fractions every other day in one application for the treatment (Arm 2). In the treatment of one BT fraction in one application, the interval between the applications was one week; while for the treatment of two continuous BT fractions every other day in one application, the interval between applications was two weeks.

### Contouring and treatment planning

The dose of the pelvic EBRT was 45–50.4 Gy, with a 1.8–2 Gy/fraction. Some patients were treated with concurrent or sequential therapy with a total lymph node boost dose of 57.5–65 Gy. The EBRT techniques included three-dimensional conformal radiotherapy (3D-CRT), intensity-modulated radiotherapy (IMRT), or volume modulated radiotherapy (VMAT). In Arm 1 and Arm 2, 52 (82.5%) and 49 (86.0%) of the patients received concurrent platinum-based chemotherapy, and 11 (17.5%) and 8 (14.0%) of the patients received radiotherapy only. All the patients received treatment by using the MRI-guided ^192^Ir HDR after-loading the therapy equipment (Micro-Selectron HDR V2), and the BT was performed after completion of the EBRT.

The implantation of the BT applicator and needle was performed as described below: the applicator was selected before the surgery, according to the disease conditions of patients. The tumor location, size, shape, para-uterine invasion, and relationship with surrounding organs were verified from the MRI images before and after the EBRT, as well as gynecological examination. The bowel preparation was performed on the day before the operation, and vaginal irrigation was performed on the day of operation. Combined intravenous and inhalation anesthesia was administered to the patients, then the patients were disinfected routinely, and the Foley urethral catheter was placed, with the balloon at the site of the vesical neck. The color ultrasound-assisted implantation of the applicator and needle was performed. The applicators used included the Utrecht interstitial Fletcher CT/MRI applicator set, interstitial ring CT/MRI applicator set, vaginal CT/MRI multi-channel applicator set, and self-made 3D-printed applicator. In patients that the para-uterine invasion has reached the pelvic wall, trans-perineal manual implantation was performed in addition to the above-mentioned applicators to meet the demands of dose distribution, and the depth of the needle was guided under the assistance of ultrasound. After the completion of the implantation, the applicator and needles were fixed. For one BT fraction in one application and two BT fractions every other day in one application, the fixation method of the applicator and needles remained the same. First, the needles were fixed on the applicator using the guiding tube, which was assembled on the applicator. Next, the applicator was filled with gauze for internal fixation, and then it was externally fixed on the patient's body with a T-shaped fixing belt. And then the rectum was pushed by the rectal pressure plate. The MRI was performed after the patient has been awakened. The application of analgesic drugs was decided based on the level of pain in patients. The analgesic drugs included were as follows: patient-controlled intravenous analgesia (PCIA), subcutaneous injection of opiates, and/or anti-inflammatory analgesics. The analgesic drugs were used in the recovery and treatment periods according to the requirements.

T2W MRI of each BT fraction was used for the delineation of target volume and OARs, as referred to in the GEC-ESTRO recommendations [4]. The high-risk clinical target volume (HR-CTV) was applied for the range of tumors that showed by the MRI and physical examinations following the EBRT, as well as the overall uterine cervix examination. The intermediate-risk clinical target volume (IR-CTV) was applied for the range of cervical cancer before the EBRT and for the extension of HR-CTV. The OARs included the bladder, small intestine, sigmoid colon, and rectum.

All the patients received the HDR-IGABT treatment at the 28 Gy/4f. The MRI was performed on the day when the operation has been completed. The patients in Arm 1 have received one BT fraction, and then the applicator or needles have been removed, while the patients in Arm 2 have received the first BT fraction. CT has been performed 16–24 h later to verify the locations of the applicator and needles. The second BT fraction was performed after confirmation.

All the patients received routine medical nursing, and stretch socks were worn to prevent deep venous thrombosis (DVT). Continuous electrocardiogram (ECG), blood pressure, and blood oxygen saturation were monitored during the waiting period. Antiemetics and anxiolytics were provided based on patient assessment. For the patients who received BT fractions every other day in one application, the segmental pressing massager was used in the waiting period, and corresponding nursing practices were also adopted during the waiting period to maintain the patients in the supine position. In addition, one doctor and two nurses were assigned for nursing and closely monitoring the continuous BT patients.

### Plan evaluation

Guided by MRI imaging, the applicator and the interstitial needles were reconstructed in the treatment planning system (TPS), and the source dwell point of the radioactive source was selected according to the shape of the target area and the relative, three dimensional positional relationship of the OARs. Dose volume histogram (DVH) parameters were used for evaluating the target volume and OARs. The equivalent dose based on linear-quadratic model in 2 Gy fraction (EQD2), with ⍺/β of 10 Gy for tumour and 3 Gy for OARs, was used to calculate the cumulative doses from EBRT and MR-IGABT. Dosimetric parameters were evaluated according to the GEC-ESTRO recommendations [4, 5].

### Follow-up and evaluation

All patients were followed up by periodical check-up in the first month after discharging from the hospital every 3 months in the first 2 years, at 6 month intervals for the next 3 years and then annually.

The BT-related toxicities were evaluated, which included pain, dizziness, nausea/vomiting, fever/infection, blood loss during the removal of applicator or needles, the DVT, and other acute toxicities. The highest numerical rating score (NRS) of the pain was recorded, and the most intense pain in the following four periods of the BT was compared: pain during the transferring from the operating room to the department (including MRI scanning process), pain during the waiting period, pain at the time of the removal of the applicator or needles, and pain in the ward after the applicator or needles were removed.

The overall survival (OS) referred to the time from the diagnosing date of cervical cancer to the death by any causes during the follow-up. Cancer-specific survival (CSS) referred to the percentage of the patients who died due to tumor-related factors in all patients. The progression-free survival (PFS) referred to the time from the diagnosing date of cervical cancer to the first recorded failure of the local control of tumor, tumor metastasis (including local metastasis and distance metastasis), or death of patients due to any causes. The local control (LC) failure referred to the tumor recurrence or enlargement of the residual lesion after treatment that was confirmed by imaging examinations and/or pathological examinations. The Common Terminology Criteria for Adverse Events (CTC-AE 5.0) was used to evaluate the incidence and severity of toxicities of the urinary system, lower digestive system, and reproduction system [6]. Toxicity of ≥ grade 3 was considered severe toxicity. Acute toxicity referred to the toxicity that occurred from the start of the radiotherapy to 90 d after the treatment was completed. Late toxicity referred to the toxicity that occurred after 90 d after the treatment was completed.

### Statistical analysis

The SPSS (v26.0) was used for statistical analysis. Descriptive analysis was performed to describe the characteristics of the patients, diseases, treatment, and treatment-related toxicities. Categorical data were compared by chi-square test or Fisher exact test. Continuous data were compared by rank-sum test. The Kaplan–Meier test was used to estimate the OS, CSS, PFS, and LC, and the Log-rank test was used to compare the differences among groups. All the statistical analyses were two-sided, and the *P* < 0.05 was considered statistically significant.

## Results

### Patients and treatment

Three patients each in Arm 1 (n = 63) and Arm 2 (n = 57) had cervical stump cancer. Thirty-nine patients received two continuous BT every other day in each application. The characteristics of the patients are shown in Table 1. OTT in Arm 2 was significantly shorter than Arm 1. The median OTT in Arm 2 was reduced to less than nine weeks, and the median OTT of the patients in Arm 2 who received both EBRT and BT at our institution was reduced to less than eight weeks. A total of 252 and 132 applications were performed in the patients in Arm 1 and Arm 2. The characteristics of the applicator and needle implantation are shown in Table 2. For the patients who received two BT fractions in one application, the IC/ISBT was used for the corresponding BT. No patients received two continuous BT fractions every other day in one application in the ICBT.

**Table 1:** Patient and tumor characteristics

Characteristic	Arm 1 (n = 63)	Arm 2 (n = 57)	*P* value
	n (%)	n (%)	
Median age	55.0 years (IQR:45.0–64.0)	54 .0 years (IQR:48.5–62.5)	0.948
FIGO stage (2009)			0.668
IB2	2 (3.2%)	3 (5.3%)	
IIA1	1 (1.6%)	4 (7.0%)	
IIA2	8 (12.7%)	6 (10.5%)	
IIB	30 (47.6%)	24 (42.1%)	
IIIA	3 (4.8%)	3 (5.3%)	
IIIB	12 (19.0%)	11 (19.3%)	
IVA	5 (7.9%)	3 (5.3%)	
IVB	2 (3.2%)	3 (5.3%)	
Histology			0.844
Squamous cell carcinoma	58 (92.1%)	54 (94.7%)	
Non-squamous cell carcinoma	5 (7.9%)	3 (5.3%)	
Concurrent chemotherapy			0.608
Yes	52 (82.5%)	49 (86.0%)	
No	11 (17.7%)	8 (14.0%)	
EBRT spot			0.949
In our hospital	35 (55.6%)	32 (56.1%)	
In other hospitals	28 (44.4%)	25 (43.9%)	
Median OTT^1^	64.0 days (IQR:57.0–85.0)	60.0 days (IQR:52.5–66.0)	**0.006**
EBRT in our hospital	59 days (IQR:54.0–63.0)	54.5 days (IQR:50.0–61.75)	**0.026**
EBRT in other hospitals	86 days (IQR:78.0–117.5)	65 days (IQR:63.0–83.5)	**0.001**

Bold values indicate statistically significance

*IQR* Inter Quartile Range, *FIGO* International federation of gynecology obstetrics, *EBRT* external beam radiotherapy, *OTT* Overall treatment time

^1^The first day of beginning EBRT to the last day finishing HDR-BT

**Table 2:** Brachytherapy treatment characteristics

Characteristic	Arm 1 (n = 252)	Arm 2 (n = 132)
	n (%)	n (%)
*Brachytherapy technique*		
IC	31 (12.3%)	5 (3.8%)
IC/IS	221 (87.7%)	127 (96.2%)
*Type of applicator*		
Utrecht	140 (55.6%)	98 (74.3%)
Ring	67 (26.6%)	26 (19.7%)
Multi-Channel	19 (7.5%)	4 (3.0%)
3D-Printed	26 (10.3%)	4 (3.0%)

*IC* intracavitary brachytherapy, *IS* interstitial brachytherapy, *IC/IS* Combined intracavitary and interstitial brachytherapy, *Utrecht* Utrecht interstitial Fletcher CT/MRI Applicator Set, *Ring* Interstitial Ring CT/MRI Applicator Set, *Multi-Channel*Vaginal CT/MRI Multi Channel Applicator Set, *3D-Printed* self-made 3D-Printed applicator

### Dosimetric data

The DVH parameters are shown in Table 3. The OARs D_2cc_ of the first and third BT fraction of patients in Arm 1 was copied and then recorded as the BT Parameters of the second and fourth BT fraction to simulate the patients in Arm1 who received two BT fractions in one application (Arm 1_simulation_). The statistical analysis showed that the OARs D_2cc_ were not significantly different between Arm 1 and Arm 1_simulation_ (Table 4).

**Table 3:** Dosimetric outcomes

Parameters	Arm 1 (n = 63)	Arm 2 (n = 57)	*P* value
	Median (IQR)	Median (IQR)	
HR-CTV D_90_, Gy	90.6 (88.25–92.21)	91.40 (86.89–93.61)	0.398
Initial HR-CTV, cm^3^	34.23 (25.78–58.80)	49.85 (37.66–75.18)	**0.003**
Bladder D_2cc_, Gy	77.44 (72.37–80.01)	82.05 (78.88–84.63)	** < 0.001**
Rectum D_2cc_, Gy	65.84 (61.14–69.59)	65.01 (60.63–69.94)	0.889
Sigmoid D_2cc_, Gy	65.72 (58.77–69.44)	67.28 (63.71–71.45)	**0.022**
Small bowel D_2cc_, Gy	64.14 (60.10–68.06)	67.64 (63.0–69.47)	**0.005**

*IQR* Inter Quartile Range

Bold values indicate statistically significance

**Table 4:** Dosimetric outcomes

Parameters	Arm 1	Arm 1_simulation_	*P* value
	Median (IQR)	Median (IQR)	
Bladder D_2cc_, Gy	77.44 (72.37–81.01)	76.77 (71.18–81.70)	0.859
Rectum D_2cc_, Gy	65.84 (61.14–69.59)	66.16 (60.87–69.20)	0.901
Sigmoid D_2cc_, Gy	65.72 (58.77–69.44)	64.5 (59.5–69.67)	0.843
Small bowel D_2cc_, Gy	64.14 (60.10–68.06)	63.57 (60.24–69.18)	0.764

*IQR* Inter Quartile Range

Bold values indicate statistically significance

### Clinical outcome

**Fig. 1:**
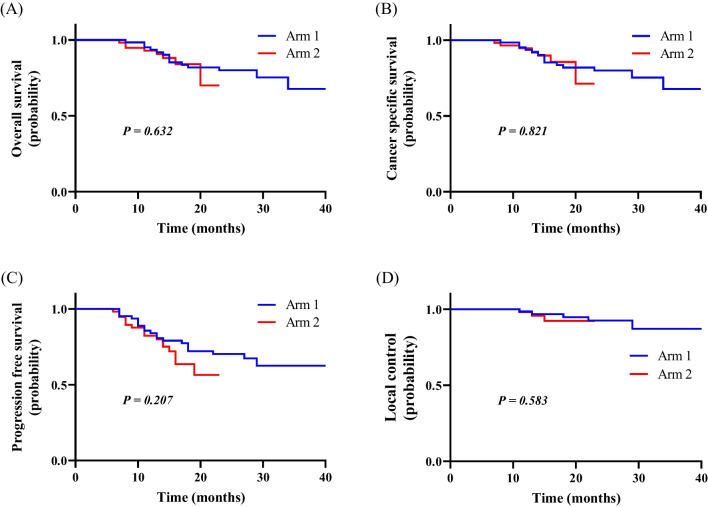
Kaplan–Meier curves of the OS (**A**), CSS (**B**), PFS (**C**), and LC (**D**) of patients in Arm 1 and Arm 2

The median (range) follow-up time in Arm 1 and Arm 2 was 23.5 (3–36) and 12.0 (5.5–20.0) months, respectively. The OS, CSS, PFS, and LC rate in Arm1 and Arm 2 was 77.8% versus 86.0% (*P* = 0.632), 77.8% vs. 87.7% (*P* = 0.821), 68.3% versus 70.2% (*P* = 0.207), and 92.1% versus 94.7% (*P* = 0.583), respectively. Figure 1 shows the Kaplan–Meier curves of the OS, CSS, PFS, and LC of patients in Arm 1 and Arm 2.

### Evaluation of the treatment-related complication

In this study, 120 patients were regrouped according to the ICBT or IC/ISBT they received and the actual BT fraction they used each application. Among the 120 patients, there were 288 treatments being conducted by performing one BT fraction per application. And 36 treatments were in the way of ICBT alone named after the ICBT group, while 252 treatments were IC/ISBT named after the IC/ISBT routine group. As for the treatment being performed by conducting two BT fractions every other day per application, named after the IC/ISBT continuous group.

The BT-related toxicities included anesthesia-related toxicities and BT implantation-related toxicities. During the BT processes, the most common anesthesia-related toxicities included nausea/vomiting and dizziness, and the BT implantation-related toxicities included pain, fever/infection, and bleeding. Among the 36 treatments in the ICBT group, two patients developed dizziness during treatment, one patient developed nausea/vomiting, and one patient had bleeding about 20 ml when the applicator was removed. No other complication occurred. The applicator implantation process of ICBT was non-invasive operation, so ICBT group was excluded from further comparisons with IC/ISBT groups.

The median (IQR) of blood loss during the removal of applicator and needles was 10 (0–20) ml versus 10 (0–27.5) ml in the IC/ISBT routine group versus the IC/ISBT continuous group (*P* = 0.458). The details of nausea/vomiting, dizziness, pain, and fever/infection in IC/ISBT routine group and IC/ISBT continuous group are shown in Table 5. The highest NRS of the pain during the waiting period and at the time of the removal of the applicator was significantly higher in IC/ISBT continuous group than IC/ISBT routine group (*P* < 0.001; Table 5). The other toxicities included transient hematuria in four patients at the time of the removal of the applicator in the IC/ISBT routine group and the DVT in one patient in the IC/ISBT continuous group. The patient with the DVT improved after treatment, but the treatment in the patient was also discontinued, and the OTT was 90 d.

**Table 5:** Treatment-related complications

Type of complication	IC/ISBT routine group (n = 252)	IC/ISBT continuous group (n = 96)	*P* value
	Mean (± SD) /n (%)	Mean (± SD) /n (%)	
Pain during transport time	1.27 ± 1.31	1.48 ± 1.37	0.204
Pain during waiting time	2.22 ± 1.84	3.02 ± 1.65	** < 0.001**
Pain during removal time	4.69 ± 1.49	5.30 ± 1.18	** < 0.001**
Pain in the ward	0.27 ± 0.46	0.33 ± 0.54	0.344
Dizziness	7 (2.8%)	2 (2.1%)	> 0.999
Nausea/Vomiting	28 (11.1%)	12 (12.5%)	0.717
Fever/Infection	20 (7.9%)	5 (5.2%)	0.378

Bold values indicate statistically significance

Analgesics were given to the patients according to the WHO Principle of Three-Step Pain Alleviation. The analgesic drugs used for the patients in the treatments are shown in Table 6. The use of analgesic drugs was significantly different between the IC/ISBT continuous group and the IC/ISBT routine group (*P* < 0.001).

**Table 6:** Application of analgesics

Analgesic drugs	IC/ISBT routine group (n = 252)	IC/ISBT continuous group (n = 96)	*P* value
	n (%)	n (%)	
No application	189 (75.0%)	7 (7.3%)	** < 0.001**
First-step	7 (2.8%)	6 (6.3%)	
Second-step	0 (0%)	0 (0%)	
Third-step	56 (22.2%)	83 (86.4%)	

Bold values indicate statistically significance

Our practices showed that the PCIA was not required for patients in the IC/ISBT routine group during the waiting period, and only 22(8.7%) PCIA were used in the IC/ISBT routine group. More PCIA was required for patients receiving continuous treatment every other day, and 65(67.7%) PCIA were used in the IC/ISBT continuous group. The 65 PCIA in IC/ISBT continuous group still needed additional analgesics during the waiting period. And the pain score of the IC/ISBT continuous group patients decreased from 3.02 ± 1.65 to 1.69 ± 0.75 (*P* < 0.001) after additional analgesics were administered.

### Acute toxicity evaluation

Of the 120 patients, the incidence of grade 2 acute toxicities of the urinary system was 3.2% and 0% (*P* = 0.497) in Arm 1 and Arm 2, respectively. The incidence of grade 2 acute toxicities of the lower digestive system was 1.6% and 1.8% (*P* = 0.999), and the incidence of grade 2 toxicities of the reproduction system was 3.2% and 5.3% (*P* = 0.667) in Arm 1 and Arm 2, respectively. No ≥ grade 3 acute toxicities were found in none of the systems. The most common acute toxicities of the urinary system included frequent urination and excessive urination at night, the most common acute toxicities of the lower digestive system included diarrhea and increased frequency of defecation, and the most common acute toxicities of the reproduction system included vaginal bleeding, vaginal inflammation (mild discomfort or pain, edema, or reddening), and increased vaginal discharge.

### Late toxicity evaluation

One patient in Arm 1 was excluded because he died of tumor metastasis within 3 months after radiotherapy, and thus 62 patients completed the follow-up for the late toxicity. The grade 3 toxicity of the lower digestive system was defecation at the frequency of ≥7 times/d in Arm 1, and intestinal obstruction in Arm 2. The grade 3 toxicity of the reproduction system was rectovaginal fistula in both Arm 1 and Arm 2. Late toxicities are showed in Table 7.

**Table 7:** Late toxicities

Toxicities	Arm 1 (n = 62)	Arm 2 (n = 57)
	n (%)	n (%)
*Urinary system*		
G0-1	61 (98.4%)	56 (98.2%)
G2	1 (1.6%)	1 (1.8%)
*Lower digestive system*		
G0-1	60 (96.8%)	55 (96.4%)
G2	1 (1.6%)	1 (1.8%)
G3	1 (1.6%)	1 (1.8%)
*Reproduction system*		
G0-1	60 (96.8%)	56 (98.2%)
G2	1 (1.6%)	0
G3	1 (1.6%)	1 (1.8%)

*G* Grade

## Discussion

This study comprehensively investigated the treatment efficacy, the BT-related toxicities, and the irradiation-related acute and late toxicities, and evaluated the applicability and safety of the MRI guided two BT fractions in one application for the treatment of cervical cancer.

The findings of this study showed that the OTT in Arm 2 was significantly shorter than Arm 1. However, the OTT in both groups in this study were relatively long, which could be associated with the following reasons: (1) several patients that could not be treated with the BT or ISBT in other hospitals were transferred to our institution after the EBRT, of which the OTT could be longer, and (2) treatment discontinuation occurred in the study in several patients due to the personal issues or acute toxicities, such as severe hematological toxicities during the EBRT. The findings of this study showed that continuous treatment every other day was the best option to shorten the OTT in patients with such conditions.

The bladder D_2cc_, sigmoid D_2cc_ and small intestine D_2cc_ were significantly different between Arm 1 and Arm 2. When comparing the treatment strategies of one BT fraction versus two BT fractions in one application, the possibility of the OARs exposed dose elevation was considered due to the continuous application of the two treatments with the high OARs exposed dose. Therefore, the Arm 1_simulation_ group was used in the study to investigate whether the strategy of two BT fractions in 1 application could increase the D_2cc_ of the OARs. Specifically, the first and third doses in Arm 1 were copied as the doses of the second and fourth treatment, which simulated that all the patients received the strategy of two BT fractions in one application. The findings showed that the D_2cc_ of the bladder, rectum, sigmoid colon and small intestine were not significantly different between Arm 1 and Arm 1_simulation_. Therefore, we speculated that two continuous treatments every other day in one application would not substantially increase the OARs exposed dose. The differences in the bladder, sigmoid D_2cc_ and small intestine D_2cc_ could be associated with the higher initial volume of the HR-CTV and the fact that the tumor was adjacent to the pelvic OARs in Arm 2.

The findings of this study showed that the treatment efficacies in Arm 1 and Arm 2 were comparable, and the LC was found in over 90% of patients in both groups. The multicentre retroEMBRACE study showed that the OTT, tumor volume, and HR-CTV D90 were important influencing factors of the tumor LC, and patients with the OTT of > 7 weeks or too large tumor volume required additional dose compensation [2]. In this study, the HR-CTV D90 dose was increased in the patients with such adverse factors to ensure the LC of the tumor. In addition, we noticed that the failures in the patients in this study were mainly due to distant metastasis, which agreed with the findings reported by previous studies [7, 8]. Therefore, it is necessary to explore other comprehensive treatment strategies to further improve the OS of the patients.

The findings of this study demonstrated that anesthesia-related toxicities in the BT were not significantly different between the IC/ISBT continuous group and IC/ISBT routine group, but the BT-related NRS of the pain was significantly different between the two groups. The pain score of the patients in the IC/ISBT continuous group during the waiting period was significantly higher than in the IC/ISBT routine group (*P* < 0.001). In addition, the use of analgesic drugs during the waiting period was also significantly different between the two groups (*P* < 0.001); specifically, the analgesic drugs used in the IC/ISBT continuous group were mainly the third-step analgesics. Due to the relatively high pain score during the waiting period in the IC/ISBT continuous group, some patients in the IC/ISBT continuous group were treated by the PCIA. And none of the patients in this study discontinued treatment owing to the pain beyond their toleration during the waiting time. The pain score in the patients in the IC/ISBT continuous group was also significantly higher than that in the IC/ISBT routine group when removing the applicator or needle, but the pain in the patients was alleviated after the applicator or needle has been removed. The NRS of the pain in both groups reduced to lower than 3 points and most was 0–1 point in several patients. Wiebe et al. [9] reported the results in 17 patients who received multiple BT fractions (IC: 82.4%, IC/IS: 17.6%) in one application, which showed that the NRS of the pain during transferring, waiting, and at the time of the removal of the applicator was 3.3 ± 2.6, 2.3 ± 2.3, and 2.7 ± 2.1, respectively. Mendez et al. [10] reported the results from 48 patients with gynecological tumors who received the ISBT 1 or 2 times, and the PCIA or oral opiates were administered during the treatment. The highest NRS of the pain was 4.7 ± 2.5 and 5.8 ± 2.3 in the two treatments, respectively. The highest pain score during the BT processes in this study was similar to the results reported by Mendez et al. [10], but the pain scores during the waiting period and at the time of the applicator or needle removal were higher than the scores reported by Wiebe et al. [9], which could be associated with the fact that most patients in this study were treated by the IC/ISBT. As many patients in this study received the IC/ISBT treatment, 64.1% of the total 348 BT fractions in both the IC/ISBT continuous group and IC/ISBT routine group were with bleeding during the removal of the applicator and needles, but no major bleeding occurred, the volume of blood loss was not significantly different between the two groups. Fokdal et al. [11] reported that in 24 patients who received the IC/ISBT, major bleeding requiring blood infusion occurred in only one patient. Bahl et al. [12] investigated 206 patients and reported major bleeding requiring blood infusion in two patients. Therefore, even using the IC/ISBT for the BT treatment, severe bleeding could occur very rarely. The overall incidence of fever/infection, nausea/vomiting, and dizziness in this study was 7.2% (IC/ISBT routine group: 7.9%, IC/ISBT continuous group: 5.2%), 11.5% (IC/ISBT routine group: 11.1%, IC/ISBT continuous group: 12.5%), and 2.6% (IC/ISBT routine group: 2.8%, IC/ISBT continuous group: 2.1%), respectively. In previous studies, the incidence of fever/infection during the BT was 2.47–14.7% [13–15]. Only very few studies reported the incidence of anesthesia-related toxicities, which varied substantially in different anesthesia methods and postoperative analgesic methods; specifically, the incidence of dizziness was 0–33.3% [16, 17], and the incidence of nausea and vomiting after the recovery from the general anesthesia was 0–21% [16, 18, 19]. Although the percentage of patients who received the IC/ISBT in this study was very high, the incidences of anesthesia-related toxicities in the BT and fever/infection were at moderate or relatively low levels, comparing with the results reported by the previous studies. For the patients who received continuous treatment every other day, the condition needing to be concerned was venous thrombosis. Dusenbery et al. [20] and Corn et al. [21] reported in their studies that the incidence of the DVT was 1.2% and 1%, respectively. Another study performed by Gupta et al. [13] also reported that the incidence of DVT was 0.37%. In this study, lower limb thrombosis and pulmonary embolism were found in one patient, who improved after the conservative treatment. Patients who received continuous treatment every other day required a longer time of immobilization than the patients in the IC/ISBT routine group; therefore, preventing the DVT and pulmonary embolism is very important for the patients receiving continuous BT every other day.

In this study, the acute toxicities and late toxicities were not significantly different between Arm 1 and Arm 2. No ≥ grade 3 acute toxicities were found in none of the systems. Totally four patients (3.4%) in Arm 1 and Arm 2 had grade 3 late toxicities, of which two were in Arm 1 and Arm 2 each. Two of the patients had cervical stump cancer; one patient had surgery to remove the bowel metastases after the radiotherapy, and the rectovaginal fistula occurred within a half year after the surgery. These findings suggested that the toxicities in this study could be the results of the joint effects of various treatment-related factors. Multiple previous studies have already demonstrated the safety of the HDR-IGABT (IC or IC/IS or IS). Additional file 1: Table S1 summarizes the late toxicities that were reported by other studies (Additional file 1). Due to the different BT doses and heterogeneities in the dose fractionations in the previous studies, the toxicities varied substantially among studies. The incidence of toxicities in this study was relatively low, which could be associated with the following reasons: (1) the follow-up time of this study was relatively short, and some late toxicities in several patients could occur after the follow-up time; (2) the irradiation-related toxicities of the OARs were dose-dependent and with the significant dose–effect relationships [22–24]. However, the patients in this study all strictly abided by the dose-volume limits of the OARs that were recommended by the guidelines, and the treatment by the IC/ISBT could have better protected the OARs. The other studies reported relatively a high incidence of severe late toxicities, and the exposure dose by the OARs was generally higher than the limit recommended by the guidelines.

In summary, the findings of this study showed that comparing with one BT fraction in one application, receiving at least one treatment of two BT fractions in one application showed no statistically significant difference in the treatment efficacy, acute toxicity, and late toxicity, but could potentially increase the tumor LC due to the shortened OTT. The treatment of two BT fractions in one application further saved the medical resources. The findings in our institution showed that comparing with the patients who received only routine treatment, the hospital stay was at least one week shorter, and the BT-related costs were about 27% lower in the patients who received at least one treatment of the continuous BT every other day. A study performed by Bajaj et al. [25] in the USA compared the doctor cost, hospital cost, and total cost between the CT guided BT with routine dose fractionations versus the MRI guided continuous BT every other day in one application, which showed that all the three costs were significantly lower in the MRI guided continuous BT every other day in one application, and the total cost was reduced by about 22.3–49%. The treatment of two BT fractions in one application reduced the times of the applicator and needle implantation, which not only avoided the trauma caused by the repeated implantation, but also reduced the times of anesthesia, and consequently reduced the incidence of anesthesia-related complications.

Comparing with the conventional one BT fraction in one application, the treatment of two BT fractions in one application required additional technical supports and human power. The technical supports included the supports from the high-quality treatment plan and high-quality nursing practices. If the coverage of the target region by the dose was not ideal in the 1st BT, the 2nd BT was not practiced continuously. The displacement of the application on the cephalocaudal direction was another especially concerning issue, which could not be avoided during the BT processes. Even in the patients receiving the routine BT therapy, the displacement of the applicator could occur in the waiting period during transferring of patients. Schindel et al. [26] performed an applicator displacement simulation and dose investigation in 20 patients who received the ICBT by the titanium tandem-and-ovoid applicator and found that the displacement on the cephalocaudal direction of ≥ 3 mm could lead to > 10% dose changes. Therefore, it is necessary to re-perform the scanning before each treatment, according to which the BT plan could be assessed. If no dislocation was found, the second BT was performed; otherwise, the dislocation should be adjusted, and the new treatment plan was decided. When performing the two BT fractions in one application strategy, additional manpower was required, and experienced doctors and nurses are also needed to prevent the development of the BT-related toxicities and to handle the toxicities in time. However, the treatment of two BT fractions in one application involved no substantial logistical burdens. These findings demonstrated that the strategy of two BT fractions in one application was an effective, safe, and medical cost-saving strategy from the aspects of both patients and hospital.

There were several limitations in this study. For instance, this study was a single-center retrospective study with a relatively small sample size and a relatively short follow-up period. The findings of the study primarily demonstrated the safety and applicability of the two MR-IGABT fractions in one application. However, the patients need to be followed up for a long time and the data need to be further investigated to verify the effects of this treatment strategy on the late toxicities of patients.

## Conclusion

The findings of this retrospective study from 120 patients with cervical cancer demonstrated that the two MR-IGABT fractions in one application strategy is a logistically applicable, safe, and effective treatment strategy that could shorten the overall treatment time and reduce the medical cost.

## Supplementary Information

**Additional file 1: Table S1.** Late toxicities of IGABT reported in studies. EBRT= external beam radiotherapy , IC= intracavitary brachytherapy, IS= interstitial brachytherapy, BT= brachytherapy, IC/ISBT= Combined intracavitary and interstitial brachytherapy, N= number, f= fraction, GI= gastrointestinal, GU= genitourinary ,G= Grade. ^a^Six 4 Gy fractions were prescribed to CTV over four days, six hours apart. ^b^BT was usually applied 3 times a week with a total of 4–5 applications planned.

## Data Availability

The datasets used and/or analyzed during the current study are available from the corresponding author on reasonable request.

## Abbreviations

IGABT: Image-guided adaptive brachytherapy
BT: Brachytherapy
EBRT: External beam radiotherapy
FIGO: International federation of gynecology obstetrics
EQD2: Equivalent dose in 2 Gy/f
GEC-ESTRO: Groupe Européen de Curiethérapie-Society for radiotherapy and oncology
OAR: Organ at risk
IC: Intracavitary
IS: Interstitial
IQR: Inter quartile range
OS: Overall survival
CSS: Cancer specific survival
PFS: Progression free survival
LC: Local control
3D-CRT: Three-dimensional conformal radiotherapy
IMRT: Intensity modulated radiotherapy
VMAT: Volume modulated radiotherapy
OTT: Overall treatment time
HR-CTV: High-risk clinical target volume
IR-CTV: Intermediate-risk clinical target volume
CTC-AE 5.0: Common terminology criteria for adverse event
HDR: High dose rate
DVT: Deep vein thrombosis
PCIA: Patient-controlled intravenous analgesia
ECG: Electrocardiogram
TPS: Treatment planning system
DVH: Dose volume histogram
GI: Gastrointestinal
GU: Genitourinary
