# Supplementary material for: The MRI-guided two adaptive brachytherapy fractions versus one adaptive brachytherapy fraction in one application for the cervical cancer: a retrospective study
Source: Radiat Oncol. 2023 Mar 6;18:46. doi: 10.1186/s13014-023-02237-0 (PMC9990264; doi:10.1186/s13014-023-02237-0)
Supplement: Supplementary file 1 — Additional file 1: Table S1. Late toxicities of IGABT reported in studies. EBRT= external beam radiotherapy , IC= intracavitary brachytherapy, IS= interstitial brachytherapy, BT= brachytherapy, IC/ISBT= Combined intracavitary and interstitial brachytherapy, N= number, f= fraction, GI= gastrointestinal, GU= genitourinary ,G= Grade. aSix 4 Gy fractions were prescribed to CTV over four days, six hours apart. bBT was usually applied 3 times a week with a total of 4–5 applications planned. [file 13014_2023_2237_MOESM1_ESM.docx]

| **Supplementary Table 1 Late toxicities of IGABT reported in studies** | | | | | | | | |
| --- | --- | --- | --- | --- | --- | --- | --- | --- |
| Study | Type | Disease site | Treatment | Total dose/dose-fraction | Brachytherapy Technique | Late GI toxicity (%) | Late lower GU toxicity (%) | Late vagina toxicity (%) |
| Pötter et al.^[1]^  （2011） | Prospective  N=156 | Cervical cancer IB_1_- IVA | EBRT ± concomitant chemotherapy＋MRI guided HDR-BT | EBRT 45-50.4Gy/1.8-2.0Gy  BT 28Gy/7Gy | 44%IC/IS | G1 12.8%  G2 7.7%  G3 1.9% | G1 6.4%  G2 4.5%  G3 1.3%  G4 1.9% |  |
| Susko et al.^[2]^  （2016） | Retrospective  N=62 | Uterine and cervical cancer | EBRT ± concomitant chemotherapy＋CT/MRI guided LDR/HDR-BT | EBRT 45-50.4Gy/1.8-2.0Gy |  |  |  | G1 24.1%  G2 24.1%  G3 9.7% |
| Rodriguez Villalba et al. ^[3]^  （2016） | Retrospective  N=25 | Cervical cancer IIA- IVB  (FIGO 2009) | EBRT＋concomitant chemotherapy＋MRI guided HDR -BT | EBRT 45-55Gy  BT 24Gy/6f ^a^ | IC/IS 100% |  | G3 2% | G3 4% |
| EMBRACE I ^[4]^  （2016） | Prospective  N=960 | Cervical cancer IA- IVA | EBRT ± concomitant chemotherapy＋MRI guided HDR/PDR-BT | EBRT 45-50Gy/1.8-2.0Gy |  | G1 20.1%  G2 6.0%  G3 1.6%  G4 0.1% |  |  |
| Mahantshetty et al.^[5]^  （2019） | Prospective  N=69 | Cervical cancer IIB- IVB | EBRT ± concomitant chemotherapy＋MRI/CT guided HDR -BT | EBRT 45-50.4Gy/1.8-2.0Gy | IC/IS 100% | G3-4 11.6% | G3-4 10.14% | G3 4.3% |
| Tiwari et al.^[6]^  （2019） | Retrospective  N=94 | Cervical cancer IIB- IVA | EBRT＋concomitant chemotherapy＋CT guided HDR -BT | EBRT 45-50.4Gy/1.8-2.0Gy  BT 25-30Gy/5f | IC/IS 55.3%  IS 44.7% | G3 1.1% | G3 1.1% | G3 1.1% |
| Vojtíšek et al. ^[7]^  （2019） | Prospective  N=61 | Cervical cancer IB_1_-IIIB | EBRT ± concomitant chemotherapy＋MRI/CT guided HDR -BT | EBRT 45Gy/25f  BT 21-35Gy/3-5f ^b^ | IC 95.1%  IC/IS 4.9% | G1 9.8%  G2 1.6%  G3 3.3%  G3 4.9% | G1 24.6%  G2 1.6%  G3 6.6% |  |
| EMBRACE I^[8]^  （2021） | Prospective  N=1153 | Cervical cancer IB- IVB | EBRT ± concomitant chemotherapy＋MRI guided HDR/PDR-BT | EBRT 45-50Gy/25-30f | IC 58.8%  IC/IS 41.2% | G1 11.1%  G2 11.4%  G3 1.4% |  |  |
| Keller et al.^[9]^（2021） | Retrospective  N=61 | Cervical cancer IB- IVB | EBRT ± concomitant chemotherapy＋MRI guided HDR -BT | EBRT 45Gy/25f  BT 23-30Gy/3-5f | IC/IS 100% | G3 5.7%（12month） | | |
| Present study  （2021） | Retrospective  N=119 | Cervical cancer  IB_2_- IVB  (FIGO 2009) | EBRT ± concomitant chemotherapy＋MRI guided HDR -BT | EBRT 45-50.4Gy/1.8-2.0Gy  BT 28Gy/4f | IC 9.5%  IC/IS 90.5% | G2 1.7%  G3 1.7% | G2 1.7% | G2 0.8%  G3 1.7% |
| EBRT= external beam radiotherapy , IC= intracavitary brachytherapy, IS= interstitial brachytherapy, BT= brachytherapy, IC/ISBT= Combined intracavitary and interstitial brachytherapy, N= number, FIGO= International federation of gynecology obstetrics, f= fraction, GI= gastrointestinal, GU= genitourinary ,G= Grade.  ^a^Six 4 Gy fractions were prescribed to CTV over four days, six hours apart.  ^b^BT was usually applied 3 times a week with a total of 4–5 applications planned. | | | | | | | | |

**Reference**

1. Pötter R, Georg P, Dimopoulos JC, et al. Clinical outcome of protocol based image (MRI) guided adaptive brachytherapy combined with 3D conformal radiotherapy with or without chemotherapy in patients with locally advanced cervical cancer. Radiother Oncol. 2011 Jul;100(1):116-23.

2. Susko M, Craciunescu O, Meltsner S, et al. Vaginal Dose Is Associated With Toxicity in Image Guided Tandem Ring or Ovoid-Based Brachytherapy. Int J Radiat Oncol Biol Phys. 2016;94(5):1099-1105. doi:10.1016/j.ijrobp.2015.12.360

3. Rodriguez Villalba S, Richart Sancho J, Otal Palacin A, Perez-Calatayud J, Santos Ortega M (2016) Development and clinical implementation of a new template for MRI-based intracavitary/interstitial gynecologic brachytherapy for locally advanced cervical cancer: from CT-based MUPIT to the MRI compatible Template Benidorm. Ten years of experience. J Contemp Brachytherapy 8(5):404-14. https://doi.org/10.5114/jcb.2016.63187

4. Mazeron R, Fokdal LU, Kirchheiner K, et al. Dose-volume effect relationships for late rectal morbidity in patients treated with chemoradiation and MRI-guided adaptive brachytherapy for locally advanced cervical cancer: Results from the prospective multicenter EMBRACE study. Radiother Oncol. 2016 Sep;120(3):412-419.

5. Mahantshetty U, Sturdza A, Naga Ch P, et al. Vienna-II ring applicator for distal parametrial/pelvic wall disease in cervical cancer brachytherapy: An experience from two institutions: Clinical feasibility and outcome. Radiother Oncol. 2019;141:123-129. doi:10.1016/j.radonc.2019.08.004

6. Tiwari R, Narayanan GS, Narayanan S, Suresh Kumar P. Long-term effectiveness and safety of image-based, transperineal combined intracavitary and interstitial brachytherapy in treatment of locally advanced cervical cancer. Brachytherapy. 2020;19(1):73-80. doi:10.1016/j.brachy.2019.10.003

7. Vojtíšek R, Sukovská E, Baxa J, et al.Late side effects of 3T MRI-guided 3D high-dose rate brachytherapy of cervical cancer : Institutional experiences. Strahlenther Onkol. 2019 Nov;195(11):972-981. https://doi.org/10.1007/s00066-019-01491-0

8. K Jensen NB, Pötter R, Spampinato S, et al. Dose-Volume Effects and Risk Factors for Late Diarrhea in Cervix Cancer Patients After Radiochemotherapy With Image Guided Adaptive Brachytherapy in the EMBRACE I Study. Int J Radiat Oncol Biol Phys. 2021;109(3):688-700. doi:10.1016/j.ijrobp.2020.10.006

9. Keller A, Rodríguez-López JL, Patel AK, et al. Early outcomes after definitive chemoradiation therapy with Vienna/Venezia hybrid high-dose rate brachytherapy applicators for cervical cancer: A single-institution experience. Brachytherapy. 2021 Jan-Feb;20(1):104-111.
